# CD133-expressing thyroid cancer cells are undifferentiated, radioresistant and survive radioiodide therapy

**DOI:** 10.1007/s00259-012-2242-5

**Published:** 2012-10-19

**Authors:** Chien-Chih Ke, Ren-Shyan Liu, An-Hang Yang, Ching-Sheng Liu, Chin-Wen Chi, Ling-Ming Tseng, Yi-Fan Tsai, Jennifer H. Ho, Chen-Hsen Lee, Oscar K. Lee

**Affiliations:** 1 Institute of Clinical Medicine, National Yang Ming University, Taipei, 11221 Taiwan; 2 Molecular and Genetic Imaging Core, NRPGM, Taipei, 11221 Taiwan; 3 School of Medicine, National Yang-Ming University, Taipei, 11221 Taiwan; 4 National PET/Cyclotron Center, Taipei Veterans General Hospital, Taipei, 11221 Taiwan; 5 Department of Biomedical Imaging and Radiological Sciences, National Yang-Ming University, Taipei, 11221 Taiwan; 6 Department of Nuclear Medicine, School of Medicine, National Yang-Ming University Medical School, Taipei, 11221 Taiwan; 7 Department of Surgery, Taipei Veterans General Hospital, Taipei, 11221 Taiwan; 8 Department of Pathology and Laboratory Medicine, Taipei Veterans General Hospital, Taipei, 11221 Taiwan; 9 Department of Pathology, School of Medicine, National Yang-Ming University, Taipei, 11221 Taiwan; 10 Institute of Pharmacology, School of Medicine, National Yang-Ming University, Taipei, 11221 Taiwan; 11 Graduate Institute of Clinical Medicine, Taipei Medical University, Taipei, 11031 Taiwan; 12 Department of Ophthalmology, Taipei Medical University-Wan Fang Medical Center, Taipei, 11696 Taiwan; 13 Center for Stem Cell Research, Taipei Medical University-Wan Fang Medical Center, Taipei, 11696 Taiwan; 14 Department of Orthopedics, Taipei Veterans General Hospital, Taipei, 11221 Taiwan; 15 Stem Cell Research Center, National Yang-Ming University, Taipei, 11221 Taiwan; 16 Department of Medical Research and Education, Taipei Veterans General Hospital, Taipei, 11221 Taiwan

**Keywords:** Thyroid cancer, CD133, Cancer stem cell, Radiotherapy, ^131^I

## Abstract

**Purpose:**

^131^I therapy is regularly used following surgery as a part of thyroid cancer management. Despite an overall relatively good prognosis, recurrent or metastatic thyroid cancer is not rare. CD133-expressing cells have been shown to mark thyroid cancer stem cells that possess the characteristics of stem cells and have the ability to initiate tumours. However, no studies have addressed the influence of CD133-expressing cells on radioiodide therapy of the thyroid cancer. The aim of this study was to investigate whether CD133^+^ cells contribute to the radioresistance of thyroid cancer and thus potentiate future recurrence and metastasis.

**Methods:**

Thyroid cancer cell lines were analysed for CD133 expression, radiosensitivity and gene expression.

**Results:**

The anaplastic thyroid cancer cell line ARO showed a higher percentage of CD133^+^ cells and higher radioresistance. After γ-irradiation of the cells, the CD133^+^ population was enriched due to the higher apoptotic rate of CD133^−^ cells. In vivo ^131^I treatment of ARO tumour resulted in an elevated expression of *CD133*, *Oct4*, *Nanog*, *Lin28* and *Glut1* genes. After isolation, CD133^+^ cells exhibited higher radioresistance and higher expression of *Oct4*, *Nanog*, *Sox2*, *Lin28* and *Glut1* in the cell line or primarily cultured papillary thyroid cancer cells, and lower expression of various thyroid-specific genes, namely *NIS*, *Tg*, *TPO*, *TSHR*, *TTF1* and *Pax8*.

**Conclusion:**

This study demonstrates the existence of CD133-expressing thyroid cancer cells which show a higher radioresistance and are in an undifferentiated status. These cells possess a greater potential to survive radiotherapy and may contribute to the recurrence of thyroid cancer. A future therapeutic approach for radioresistant thyroid cancer may focus on the selective eradication of CD133^+^ cells.

## Introduction

Thyroid cancer is the most common endocrine cancer and is still increasing in incidence [1, 2]. Papillary thyroid carcinomas (PTC) and follicular thyroid carcinomas (FTC), often referred to together as well-differentiated thyroid cancer (WDTC), account for more than 90 % of all thyroid cancers. Generally, PTC and FTC are two of the most curable cancers: they have a favourable prognosis with 10-year overall survival rates of 93 % and 85 %, respectively [3]. Anaplastic thyroid cancer (ATC), or so-called undifferentiated thyroid cancer (UTC), is a rare, aggressive and lethal malignancy with rapid progression. Patients with ATC have a median survival time of no more than 6–8 months [4, 5]. In terms of morphological appearance and biological behaviour, poorly differentiated thyroid cancer (PDTC) represents intermediate entities in the progression from WDTC to ATC, and has an intermediate prognosis [4].

Conventional management of patients with thyroid cancer involves surgical removal of the tumour followed by radioiodine ablative therapy. The efficacy of radioiodine ablative therapy depends upon the uptake of ^131^I by local residual or metastatic lesions. Several distinct molecules cooperate in the process of thyroid hormone synthesis leading to iodide accumulation and retention in the cells. These molecules include sodium iodide symporter (NIS) that pumps iodide into the cells and thyroperoxidase (TPO) that oxidizes iodide to iodine and iodinates thyroglobulin (Tg) [6]. Expression of these genes is lower in PDTCs than in WDTCs and cannot be detected in ATCs [7, 8], and is thought to be the major cause of the failure of ^131^I therapy.

The cancer stem cell (CSC) model is based on the hypothesis that there is a subset of cancer cells possessing the ability to self-renew and to generate differentiated progeny. These cells, which resemble normal stem cells and are hierarchically more primitive, express stem cell-related marker genes and are highly resistant to standard chemotherapeutic agents as well as radiation [9, 10]. CD133, also known as prominin-1, is a five-transmembrane domain glycoprotein specifically expressed on the surface of haematopoietic stem and progenitor cells [11]. Previous studies have shown that CD133 is a marker of CSCs in different types of cancer including brain tumours [12], colon cancer [13, 14] and melanoma [15]. In terms of the relationship between CD133 and thyroid cancer, Zito et al. found that two human ATC cell lines, ARO and KAT-4, contain subpopulations of CD133^+^ cells which exhibit stem cell-like features such as rapid proliferation, the ability of self renewal and to form colonies, and increased resistance to chemotherapy-induced apoptosis in vitro [16]. Friedman et al. reported that CD133^+^ ATC cells are solely responsible for tumour growth in immunodeficient mice in response to thyroid-stimulating hormone (TSH) [17]. These results strongly suggest that CD133^+^ cells in thyroid cancers represent a more undifferentiated population and possess several stem cell phenotypic characteristics. However, it is not known whether these CD133^+^ cells in thyroid cancers, even in WDTC, are the major source of cells that survive ^131^I treatment and thus potentially contribute to recurrence or metastasis of thyroid cancer.

To date, as far as we know no information is available on whether the presence of CD133^+^ cells correlates with failure of ^131^I treatment of thyroid cancer. The aim of this study was to investigate the existence of CD133^+^ cells in thyroid cancer and to explore their role in the radiation treatment of thyroid cancer. We hypothesized that the CD133^+^ populations in thyroid cancer cells are responsible for the resistance to radiation treatment of thyroid cancers.

## Materials and methods

### Cell culture

The thyroid cancer cell lines used in this study included cells lines of the follicular type (WRO and CGTH), the papillary type (CG3) and the anaplastic type (ARO). The CGTH and CG3 cell lines were kindly provided by Dr. Jen-Der Lin (Chang Gung Memorial Hospital, Taipei, Taiwan). All cell lines were cultured on RPMI-1640 (Sigma-Aldrich, St. Louis, MO) supplemented with 10 % fetal bovine serum (FBS; Invitrogen, Grand Island, NY). Tissue samples obtained from patients diagnosed with PTC after they had provided informed consent were used for primary culture of PTC cells. Most of the patients had undergone total thyroidectomy. The collected tissues were minced and washed with phosphate-buffered saline (PBS; Sigma-Aldrich, St. Louis, MO). Then the minced tissues were digested with collagenase II at a final concentration of 2 mg/ml at 37 °C for 30 min. After centrifugation, the cells were cultured in F-12K medium (Invitrogen, Grand Island, NY) supplemented with 10 % FBS, hydrocortisone (5 × 10^−6^ M), TSH (5 × 10^−3^ IU/ml), insulin (10 µg/ml) and glycyl-l-histidyl-l-lysine acetate (10 ng/ml) (Sigma-Aldrich, St. Louis, MO) [18]. This study was conducted under approval of the institutional review board.

### Cell staining, flow cytometry analysis and cell isolation

The CD133^+^ cell population was isolated by fluorescence-activated cell sorting (FACS) or magnetic selection. For FACS isolation, the cells were washed with PBS containing 0.1 % FBS and 2 mM EDTA, and then incubated with antibody against CD133/1 conjugated with phycoerythrin (PE; Miltenyi Biotec, Bergisch Gladbach, Germany) on ice in the dark for 20 min. After the cells had been washed with PBS, the CD133^+^ and CD133^−^ populations were isolated by FACS using an Aria cell sorter (BD Biosciences). For magnetic selection, the cells were labelled with 0.5 mL micromagnetic beads conjugated with CD133/1 antibodies and isolation was carried out using a MACS column and isolator (Miltenyi Biotec). For annexin V and CD133 double staining, cells were trypsinized and washed with 1× binding buffer and then incubated with FITC annexin V (BD Pharmingen) for 15 min at room temperature in the dark. After annexin V staining, CD133-PE staining was performed on the same cells following the procedure described above.

### Clonogenic radiation survival assay

Cultured cells were trypsinized, counted and plated. After 24 h of incubation, the cells were irradiated using a ^137^Cs source (model 143-68 blood product irradiator; JL Shepherd and Associates, San Fernando, CA ) to give different doses. After 7–14 days of incubation, colonies were fixed with 3.7 % formaldehyde and stained with crystal violet. Colonies consisting of >50 cells were counted. The surviving fraction at each dose was calculated following the equation: number of colonies/(number of cells seeded × plating efficiency), where plating efficiency is (number of formed colonies/number of cells seeded). The surviving fraction at each dose point was determined in triplicate and the results are shown as means ± SD.

### Real-time quantitative polymerase chain reaction

Total RNA was extracted from cells or tumour tissue using Trizol reagent (Invitrogen, Grand Island, NY) and was then reverse-transcribed into first strand cDNA. All reactions were prepared by mixing 50 ng first strand cDNA with TaqMan Fast Universal PCR Master Mix (Applied Biosystems, Foster City, CA) and corresponding primers. Real-time quantitative polymerase chain reaction (PCR) was carried out using a StepOnePlus Real-Time System (Applied Biosystems, Mannheim, Germany) with the following programme: activation at 95 °C for 20 s followed by 50 cycles of 95 °C for 1 s and 60 °C for 20 s. All the intron-spanning primers were designed at the Universal ProbeLibrary Assay Design Center (Roche Applied Science; http://www.roche-applied-science.com/sis/rtpcr/upl/ezhome.html). The primer sequences and probe numbers from Universal ProbeLibrary were as follows: NIS (forward tctctcagtcaacgcctctg; reverse gcgtccattcctgagctg; probe no. 52), Tg (forward tggagctttcagccagactc; reverse ccattctggtcacattgcag; probe no. 6), TPO (forward cagcccatggacattactcc; reverse ttgcaagaaggcctcgtatt; probe no. 10), TSH receptor (TSHR) (forward cctgacagttattgacaaagatgc; reverse ctggtttgagacacgtccag; probe no. 25), thyroid-specific transcription factor 1 (TTF1) (forward ctcggactacggcaccat; reverse gcgtcctctcaccaggtc; probe no. 39), paired box gene 8 (Pax8) (forward tgcctcacaactccatcaga; reverse aggtctgccattcacaaagg; probe no. 33), Oct4 (forward gtggagagcaactccgatg; reverse tctgcagagctttgatgtcc; probe no. 78), Nanog (forward atgcctcacacggagactgt; reverse agggctgtcctgaataagca; probe no. 69), Sox2 (forward ttgctgcctctttaagactagga; reverse ctggggctcaaacttctctc; probe no. 35), Lin28 (forward gaagcgcagatcaaaaggag; reverse gctgatgctctggcagaagt; probe no. 23), Glut1 (forward ggttgtgccatactcatgacc; reverse cagataggacatccagggtagc; probe no. 67).

### Animal study and in vivo microSPECT/CT imaging

All animal manipulation procedures were in accordance with the institutional animal welfare guidelines. ARO cells that stably express human *NIS* gene (ARO-hNIS) had been established in our previous work [19]. A total of 5 × 10^5^ ARO-hNIS cells were implanted subcutaneously into the right shoulder of 6-week-old severe combined immunodeficiency (SCID) mice. Two weeks later, when the diameter of the tumours was about 5 mm, the mice were ^131^I treated. Specifically, five mice were injected intraperitoneally with 37 MBq (1 mCi) of ^131^I and another five mice were injected with the same volume of normal saline. Tumour volume was measured and recorded on days 0, 4, 7 and 11. On day 11, tumours were removed after the mice had been killed by injection of an overdose of pentobarbital. The tumour tissues were then minced and total RNA was extracted using Trizol reagent. Gene expression was analysed as described above.

For dosimetric calculation, two SCID mice with developed ARO parental and hNIS-expressing tumours (about 500 mm^3^) on the left and right shoulders, respectively, were subjected to scintigraphic imaging after administration of 1.85 MBq (50 µCi) of ^131^I [19]. Serial images were acquired for 20 min at 1 h, 3 h, 5 h, 7 h, 14 h, 28 h and 48 h after injection using a gamma camera (ECAM; Siemens, Hoffman Estates, IL). Accumulated event counts in a selected tumour area on each image were converted to radioactivity values (megabecquerels) by calibrating the gamma camera with defined activities. No corrections were made for scatter, attenuation or recovery. Time–activity curves of both ARO parental and hNIS-expressing tumours were fitted to an exponential function and the accumulation of radioactivity was integrated according to the fitted function over a time interval from 0.5 to 60 h. Integrated radioactivity in hNIS-expressing and parental tumours were 1.69 and 0.36 MBq•h, respectively. Dosimetric calculation was based on the assumption that the tumours were spherical with a density of 1 g/ml and homogeneous radionuclide distribution. The absorbed dose of the tumours was calculated following the MIRD (Medical Internal Radiation Dose) schema using OLINDA 1.0 software.

In vivo animal SPECT/CT imaging was performed on a triple-head CZT gamma camera equipped with a built-in CT component (Triumph-SPECT; Gamma Medica, Northridge, CA) and a high-resolution multiple pinhole collimator (focal length 75.0 mm, aperture diameter 1.0 mm). This system applied circular scanning protocols for both SPECT and CT acquisition, with a translation stage invariable axial imaging range. Before imaging, each mouse was injected with 1.85 MBq (50 µCi) of ^123^I via a tail vein. At 60 min after injection, the mice were fixed in a prone position on the imaging table under gaseous anaesthesia (2 % isoflurane and 98 % oxygen) and imaged with X-ray CT with a current of 0.5 mA and voltage of 80 kVp. For SPECT imaging, 32 projections (28 s per projection, radius of rotation 4 cm, field of view 5.28 cm) were acquired in a 180° orbit. The energy window was set at 159 keV peak with a 20 % band. The SPECT image dataset was then reconstructed using the ordered-subset expectation maximization algorithm with standard-mode parameters as provided by the manufacturer. No scatter or attenuation correction was applied to the reconstructed images. The images were viewed and presented using AMIDE software (free software provided by Source Forge).

### Statistical analysis

Two-tailed Student’s *t* test was performed for the statistical analysis. A *p* value <0.05 was considered statistically significant.

## Results

**Fig. 1:**
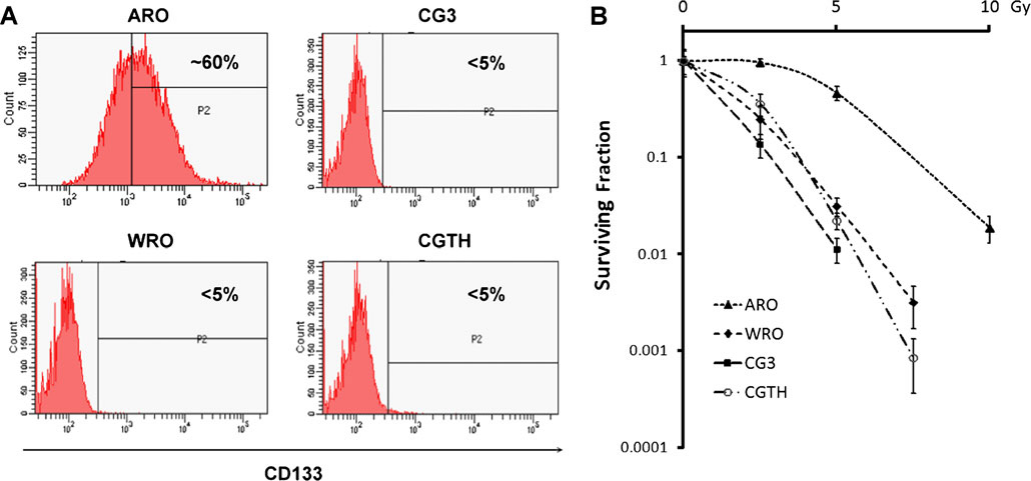
CD133^+^ population and radiosensitivity in thyroid cancer cell lines. **a** Four thyroid cancer cell lines, ARO, WRO, CG3 and CGTH were analysed to explore their CD133^+^ population using flow cytometry. CD133^+^ populations were determined according to the binding of the isotype-matched control in each cell line. Similar results were observed in two independent experiments. **b** Radiosensitivity was assessed by clonogenic survival assays after the cells had been γ-irradiated at different doses. The surviving fractions are shown as means ± SD (*n* = 3)

It has been reported that ARO cells contain 57 % CD133^+^ cells, and these have been demonstrated to act as CSCs in thyroid cancer [16]. We wondered if the percentage of CD133^+^ population varies across thyroid cancer cell lines with various levels of differentiation. Four thyroid cancer cell lines were analysed in this study, namely WRO, CGTH (FTC), CG3 (PTC) and ARO (ATC). Using flow cytometry, the CD133-expressing populations in these lines were assessed. The anaplastic type ARO line comprised about 60 % CD133^+^ cells, while the other three lines representing WDTC had less than 5 % of CD133^+^ cells (Fig. 1a). We assessed the sensitivity to irradiation of the various cell lines with different CD133^+^ populations. A clonogenic radiation survival assay was carried out by irradiating cells with different doses and counting the number of colonies formed after irradiation. The radiation survival curves of the four cell lines showed that ARO cells exhibited a markedly higher radioresistance than the other three WDTC lines (Fig. 1b).

**Fig. 2:**
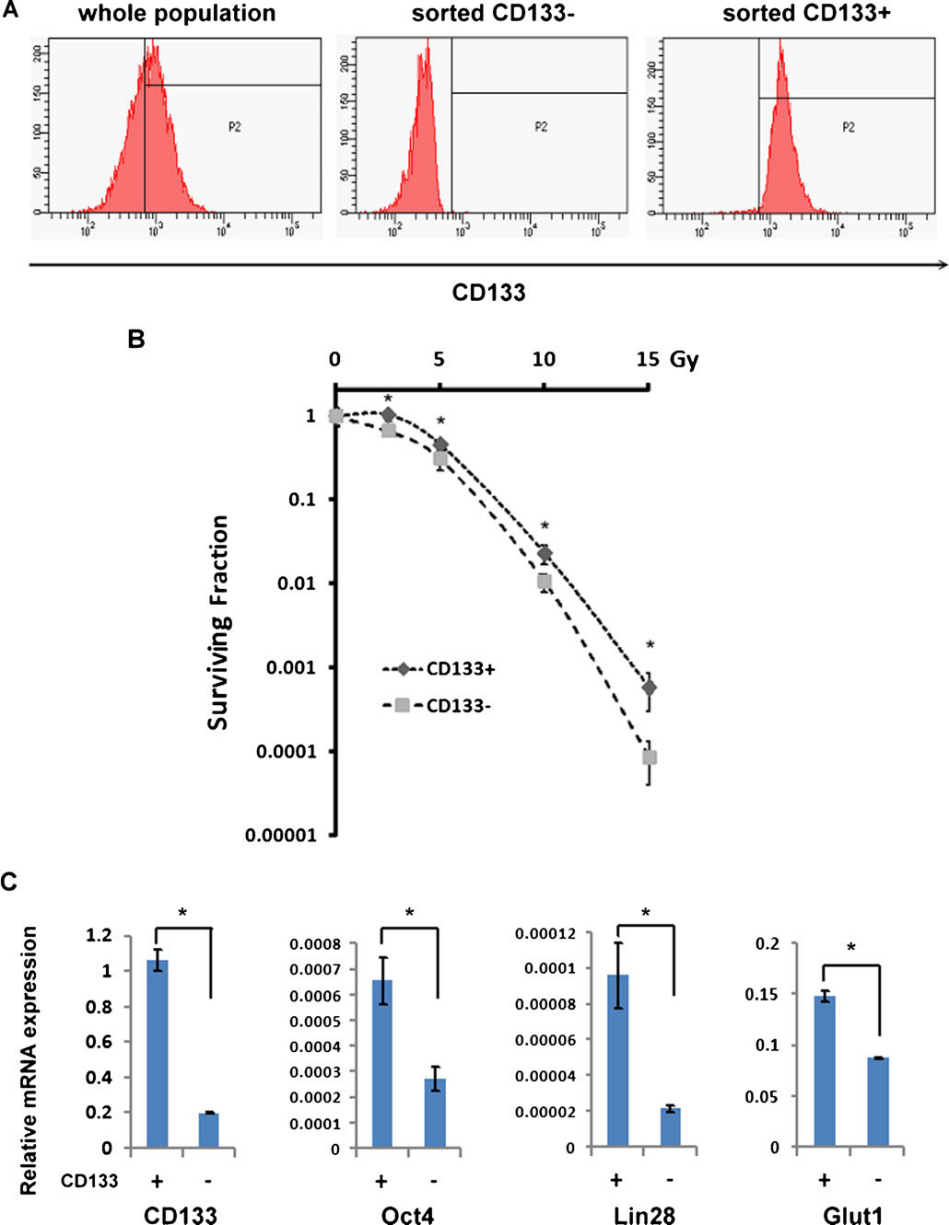
Radiosensitivity and gene expression of CD133^+^ and CD133^−^ populations of the ARO thyroid cancer cell line. **a** The CD133^+^ and CD133^−^ populations were isolated by FACS. The proportion of CD133^+^ cells was determined by flow cytometry before and after cell sorting. **b** The surviving fractions of isolated CD133^+^ and CD133^−^ populations were assessed by clonogenic assays after irradiation. The surviving fractions are shown as means ± SD (*n* = 3) **c** The sorted CD133^+^ and CD133^−^ cells were analysed for *CD133*, *Oct4*, *Lin28* and *Glut1* mRNA expression using real-time quantitative PCR. Expression of each gene was normalized to that of *GAPDH* mRNA and is presented as 2^−ΔCt^. The relative mRNA expression levels are shown as means ± SD (*n* = 2)

As CD133-expressing cells are the putative CSCs in these thyroid cancer cell lines, we speculated that the CD133^+^ population contributes to the radioresistance of bulk cancer cells. CD133^+^ and CD133^−^ cells were isolated from ARO cells (Fig. 2a) and then subjected to a clonogenic radiation survival assay. As shown in Fig. 2b, CD133^+^ cells were significantly more radioresistant than CD133^−^ cells, indicating that these two populations of ARO cells possessed different levels of radiosensitivity. CD133^+^ cells also expressed a higher level of *Oct4* gene, which is known to be responsible for the maintenance of the pluripotency and the proliferation of embryonic stem (ES) cells. The expression of *Lin28*, another marker of ES cells, was also higher in CD133^+^ cells than in CD133^−^ cells. Furthermore, CD133^+^ cells showed higher expression of *Glut1*, which indicates dedifferentiation and an unfavourable prognosis of thyroid cancer [20, 21]. Thus, CD133^+^ cells represent a relatively primitive and more radioresistant population than CD133^−^ cells within the same bulk cancer cell population, suggesting that radiotherapy would result in uneven cell killing.

**Fig. 3:**
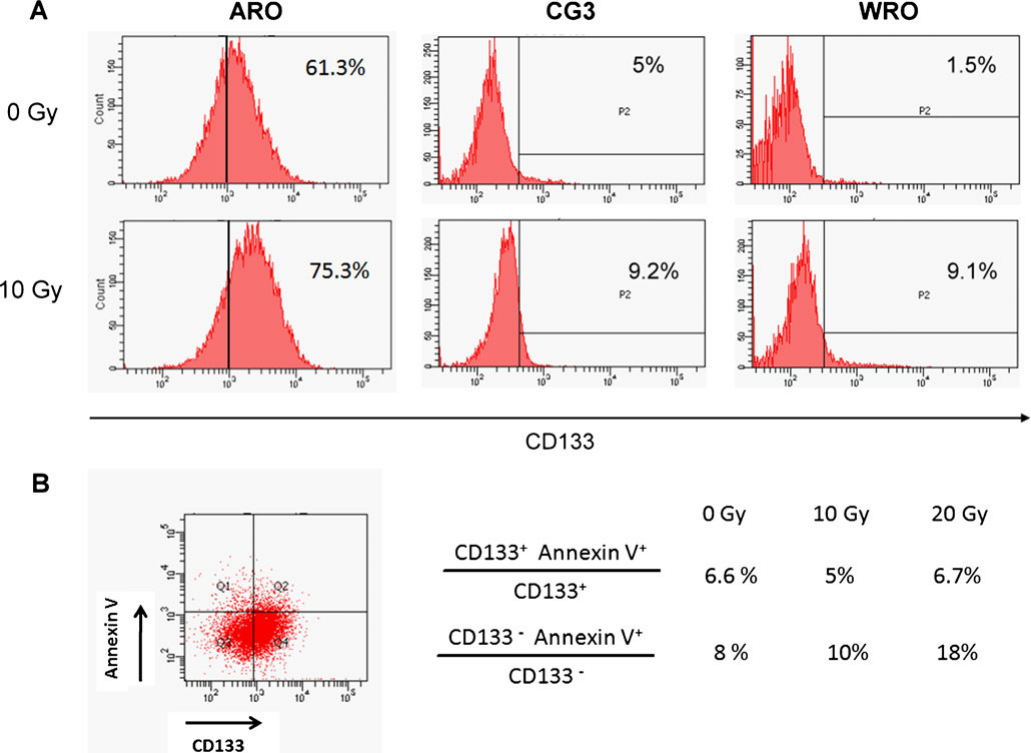
Effect of radiation on the CD133 populations. **a** ARO, WRO and CG3 cells were analysed for CD133^+^ populations by flow cytometry before and 48 h after γ-irradiation. **b** Apoptosis in the CD133^+^ and CD133^−^ populations was determined by analysis of annexin V binding. Similar results were observed in two independent experiments

To investigate whether radiation treatment of cancer cells affects the CD133^+^ population, ARO, CG3 and WRO cells were irradiated and then analysed by flow cytometry to assess their CD133 expression. At 48 h after γ-irradiation at 10 Gy, the CD133^+^ population had increased by 14 % (61 % to 75 %), 4 % (5 % to 9.2 %) and 7.5 % (1.5 % to 9 %) in ARO, WRO and CG3 cells, respectively (Fig. 3a). We further analysed the annexin V binding of these cells to elucidate whether CD133^+^ or CD133^−^ cells are more susceptible to apoptosis after radiation treatment. At 48 h after irradiation with 0, 10 or 20 Gy γ-radiation, ARO cells were double-stained with CD133-PE and annexin V-FITC and analysed by flow cytometry. Among the CD133^+^ cells, the CD133^+^/annexin V^+^ subpopulation remained relatively unchanged after γ-irradiation: 6.6 %, 5 % and 6.7 % after irradiation with 0, 10 and 20 Gy, respectively. Among the CD133^−^ cells, the CD133^−^/annexin V^+^ subpopulation showed quantitative increases after γ-irradiation: 8 %, 10 % and 18 % increases after irradiation with 0, 10 and 20 Gy, respectively (Fig. 3b). The CD133^+^ cells were more resistant to apoptosis than the CD133^−^ cells after radiation treatment which may the enrichment of the CD133^+^ population.

**Fig. 4:**
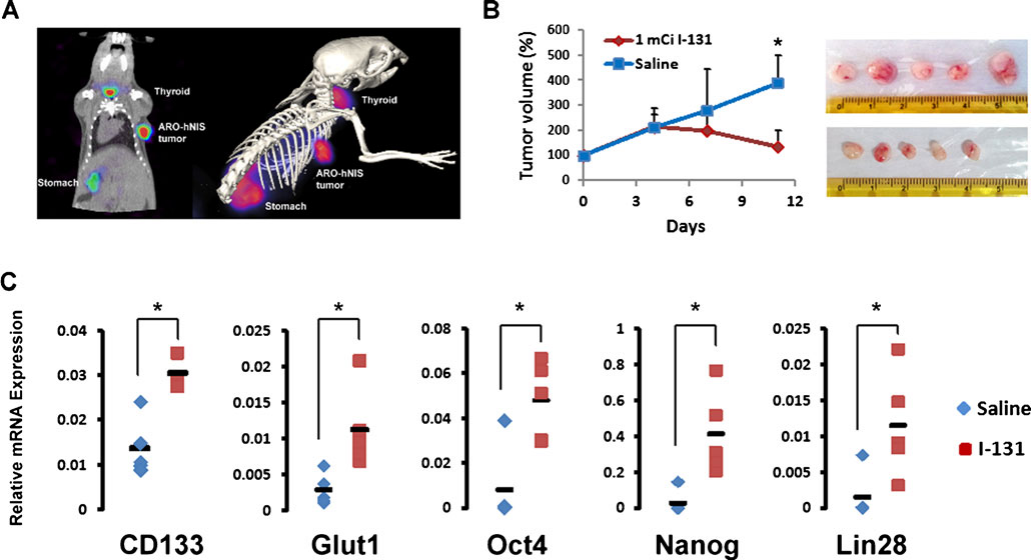
Gene expression profile after in vivo ^131^I treatment of an ARO-hNIS tumour. **a** ARO cells stably expressing hNIS (ARO-hNIS, established in our previous study) were injected subcutaneously into the right shoulder of SCID mice. Radioiodine uptake by the ARO-hNIS tumours was verified by intravenous injection of 1.85 MBq (50 µCi) ^123^I followed by in vivo imaging using a microPET/SPECT/CT. **b** The mice bearing the ARO-hNIS tumours received an intraperitoneal injection of 37 MBq (1 mCi) ^131^I. Mice injected with normal saline formed the control group. Each group consisted of five mice. Tumour volume was measured on various days. On day 11, the mice were killed and the tumours were excised for subsequent gene expression analysis. **c** After tumours had been minced and digested, total RNA was isolated, reverse-transcribed and the expression levels of *CD133*, *Glut1*, *Oct4*, *Nanog* and *Lin28* mRNA were determined by real-time quantitative PCR using designed primers. Expression of each gene was normalized to that of *GAPDH* mRNA and is presented as 2^−ΔCt^. The relative mRNA expression levels are shown as means ± SD (*n* = 2)

Radiation treatment also preferentially induced more apoptosis in CD133^−^ cells than in CD133^+^ cells in vitro. In order to elucidate whether clinical ^131^I treatment of thyroid cancer has a similar effect, we used ARO-hNIS, an ARO cell line that stably expresses the human hNIS gene [19] in an in vivo study. ARO-hNIS cells were injected subcutaneously into the shoulder of 6-week-old mice. In vivo radioiodide uptake by the ARO-hNIS tumours was assessed using a microPET/SPECT/CT imager after the mice had been injected with 1.85 MBq (50 µCi) ^123^I. The SPECT/CT images clearly showed accumulation of ^123^I in tumour (Fig. 4a), indicating that the re-expression of hNIS in ARO cells efficiently restores the ability to take up ^131^I. Based on this result, mice were treated with an intraperitoneal injection of 37 MBq (1 mCi) ^131^I, while a control group were treated with the same volume of normal saline. According to the dosimetric calculation, hNIS-expressing ARO tumours treated with ^131^I received a dose of approximately 0.195 Gy/MBq, which was five times higher than that received by ARO parental tumours (0.0421 Gy/MBq). Tumour growth was recorded and on day 11 after ^131^I administration, the volumes of the tumours in the experimental group were significantly reduced compared to those in the saline-treated group (Fig. 4b). This efficient inhibition of tumour growth confirms the killing effect of ^131^I accumulated in the tumour cells. The tumours were then excised and total RNA was extracted for gene expression analysis. The expression levels of *CD133*, *Glut1*, the ES cell-related regulator *Oct4*, *Nanog* and *Lin28* were elevated in ^131^I-treated tumours compared to the levels in saline-treated tumours (Fig. 4c). The results of this in vivo study strongly suggests that the more primitive of the CD133^+^ cells have a greater ability to survive radiation treatment.

**Fig. 5:**
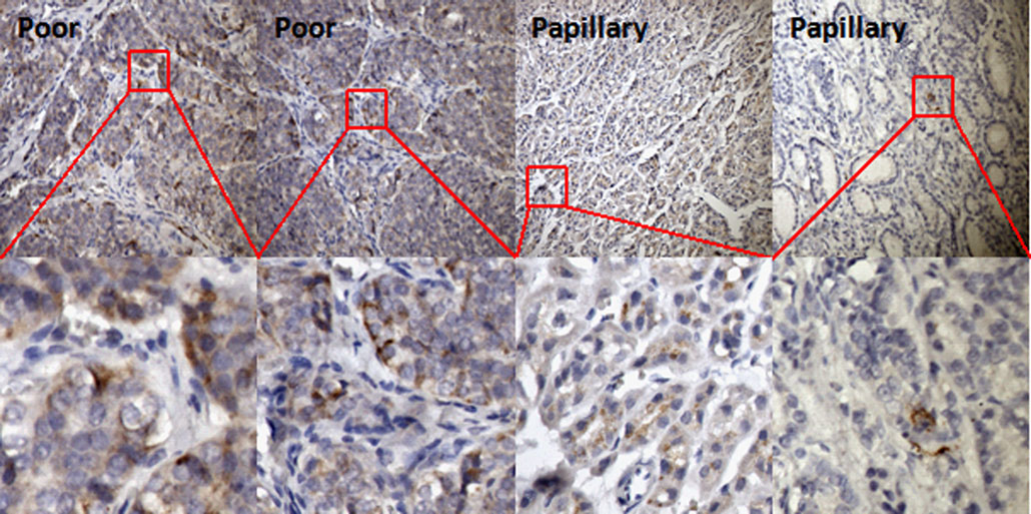
Immunohistochemical staining for CD133 in clinical. CD133 expression was detected in paraffin-embedded sections of two UTCs (*Poor*) and two PTCs (*Papillary*)

**Fig. 6:**
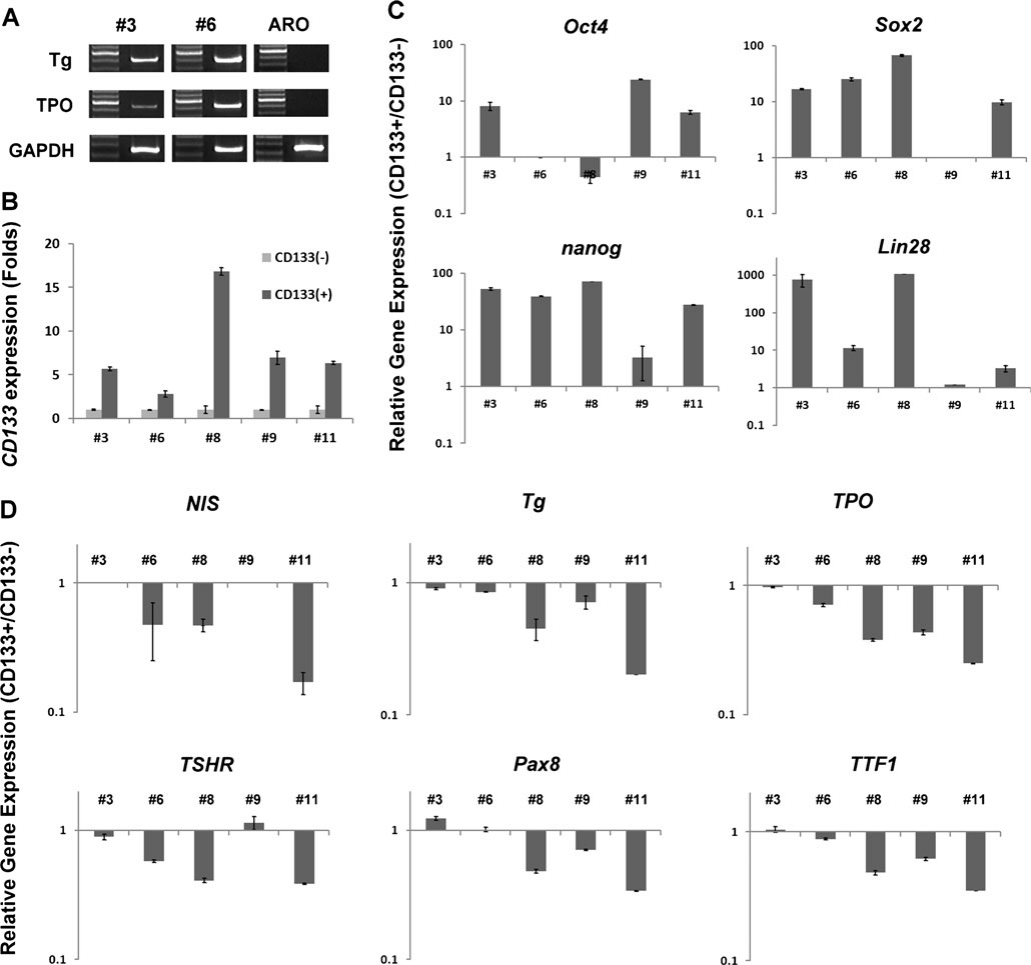
Gene expression profiles of CD133^+^ and CD133^−^ cells from primarily cultured PTCs. **a** Clinical PTCs removed from patients were minced, digested and then subjected to primary culture. After total RNA isolation, the thyroid origin of the cells was confirmed by examining *Tg* and *TPO* expression using RT-PCR and ethidium bromide-stained agarose gel electrophoresis. ARO cells are known to lack thyroid-specific gene expression and served as the negative control. **b** CD133^+^ and CD133^−^ populations isolated from primarily cultured cells by FACS were subjected to total RNA isolation and first-strand cDNA transcription. Expression levels of *CD133* mRNA by the isolated populations were analysed by real-time quantitative PCR and normalized to that of *GAPDH* mRNA. **c** Expression levels of *Oct4*, *Nanog*, *Lin28* and *Sox2* were assessed in the CD133^+^ and CD133^−^ cell populations by real-time quantitative PCR. **d** Expression levels of *NIS*, *Tg*, *TPO, TSHR, TTF1* and *Pax8* were assessed in CD133^+^ and CD133^−^ cell populations by real-time quantitative PCR. All experiments were performed at least in duplicate and normalized to *GAPDH* mRNA expression. Data shown are the ratios of gene expression levels in the CD133^+^ population to those in the CD133^−^ population

Cells with heterogeneous phenotypes in a bulk tumour are commonly seen in various types of cancer. We have demonstrated that a CD133^+^ population exists in different proportions in different thyroid cancer cell lines and this CD133^+^ population shows higher radioresistance than the CD133^−^ population. We further sought to determine whether CD133 was expressed in clinical thyroid cancers. We analysed paraffin-embedded tissues from UTC and PTC by immunohistochemical staining using anti-human CD133 antibody. CD133 expression was detectable in these types of thyroid cancer, and UTCs showed more positive staining than PTCs (Fig. 5). However, CD133 expression was also detectable in PTCs, even though this type of thyroid cancer is relatively well differentiated. Furthermore, cells from primary cultures of fresh resected PTCs expressed the thyroid-specific genes *NIS* and *TPO* after culture for a number of passages (Fig. 6a). CD133^+^ and CD133^−^ cells were separated from the PTC primary cultures and the gene expression profile was analysed. *CD133* gene expression levels in the separated CD133^+^ and CD133^−^ populations from five primary lines are shown in Fig. 6b. CD133 expression was 4- to about 16-fold higher in the CD133^+^ population than in the CD133^−^ population. The CD133^+^ population also showed higher expression levels of various genes related to ES cells, including *Oct4* (CD133^+^ about 8- to 24-fold higher), *Nanog* (about 3- to 71-fold higher), *Lin28* (about 1- to 1,000-fold higher) and *Sox2* (about 10- to 68-fold higher) (Fig. 6c). Thyroid-specific gene expression was lower in the CD133^+^ population than in the CD133^−^ population, including *NIS* (CD133^−^ about 2- to 5-fold higher), *Tg* (about 1- to 5-fold higher), *TPO* (about 1- to 4-fold higher), *TSHR* (about 0.8- to 2.5-fold higher), *Pax8* (about 0.8- to 3-fold higher) and *TTF1* (about 1- to 3-fold higher) (Fig. 6d). These results clearly indicate the existence of a CD133^+^ population in primary PTCs which are more undifferentiated.

## Discussion

^131^I radiotherapy is an important part of thyroid cancer management and its efficacy is closely relevant to future recurrence and metastasis. Although CD133^+^ cells have been proposed as thyroid CSCs, their role in radioiodide therapy of thyroid cancer has not been explored. The present study unequivocally demonstrated that CD133^+^ thyroid cancer cells are able to survive radiotherapy and these surviving cells may potentiate future recurrence. CD133^+^ thyroid cancer cells had a higher radioresistance than CD133^−^ cells, and the population of CD133^+^ thyroid cancer cells was enriched after radiation treatment. Also, these cells showed higher expression of ES cell-related genes (*Oct4*, *Nanog* and *Lin28*) and lower expression of thyroid-specific genes (*NIS*, *Tg*, *TPO* and *TSHR*) than CD133^−^ cells. This indicates that these CD133^+^ cells resemble stem cells and may possess higher tumour-initiating ability.

It has been reported that parallel expression of various thyroid-specific genes (*NIS*, *Tg*, *TPO*, *TSHR*, *TTF1* and *Pax8*) is inversely correlated with differentiation and malignant phenotypes [22, 23]. This gene expression pattern has been found in clinical and pathological samples and indicates a stepwise progression of thyroid cancer from WDTC to UTC or ATC [24]. An area of PTC in ATCs has been shown to undergo dedifferentiation, and this process has been reported to be correlated with gaining an additional *p53* mutation in *BRAF*-mutated PTCs [25, 26]. However, how the tumour cells dedifferentiate and how these genes mutate during thyroid cancer progression remains unknown. The recently proposed CSC concept offers a new insight into cancer progression. Several studies have demonstrated the existence of thyroid CSCs marked by CD133 expression [16, 17], by aldehyde dehydrogenase expression [27] or as a side population in flow cytometry [28]. Together with our results, these cells showing gene expression related to an undifferentiated status are resistant to radiation and chemotherapy drugs and exhibit stem cell-like behaviour. However, these putative thyroid CSCs have only been demonstrated to exist as a more primitive population within heterogeneous thyroid cancer. Whether these CSCs participate in the dedifferentiation during thyroid tumour progression needs to be further addressed in the future.

In this study, we demonstrated that the expression levels of several genes in the ARO-hNIS tumours were altered following ^131^I treatment. ARO-hNIS tumour growth was efficiently inhibited by ^131^I treatment, following which the expression levels of ES cell regulators/markers as well as *Glut1* were upregulated. We attribute this phenomenon to the selection effect of ^131^I radiation within the heterogeneous cancer cells which resulted in the greater survival of a radioresistant population, namely CD133^+^ cells in this study. CD133^+^ populations, both in thyroid cancer cell lines and in primary cultured PTC cells (data not shown), were enriched after irradiation. This enrichment was due to the higher radioresistance of a group of cells primarily comprising CD133-expressing cells. In addition to the selective killing effect, ^131^I treatment has also been reported to induce dedifferentiation of thyroid cancer cells. After pretreatment with ^131^I, FTC-133, a DCT cell line showed decreased ability to accumulate radioiodide, which was correlated with the downregulation of NIS, Tg, TPO and TSHR expression [29]. Taken together, despite the effectiveness of ^131^I therapy in thyroid cancer, its adverse effects, selective killing and induction of dedifferentiation should be generally considered in the management of thyroid cancer.

Dedifferentiation of thyroid cancer is characterized by a reduction or loss of thyroid-specific gene expression. As a result, the tumours become insensitive to radioiodide therapy due to a loss of radioiodide uptake and accumulation in the cells [30]. In the present study, after in vitro γ-irradiation, isolated CD133^+^ cells showed a higher survival rate than isolated CD133^−^ cells. Also, among the bulk cells, the CD133^+^ population was enriched after γ-irradiation due to a lower rate of apoptosis. These results indicate that CD133^+^ cells have a higher innate radioresistance in addition to their diminished ability to take up and accumulate iodide. In our in vivo animal ARO-hNIS tumour model, ^131^I treatment efficiently inhibited tumour growth and led to a significant elevation in CD133 expression compared to saline treatment. This result clearly demonstrates the enrichment of CSCs after ^131^I radiation and also implies the possible role of these cells in future recurrence and metastasis.

CD133-expressing cells were found in thyroid cancer cell lines, thyroid cancer tissues and primarily cultured PTCs, suggesting that CD133 expression contributes to the heterogeneity of thyroid cancer. Purified ARO CD133^+^ cells recapitulated the parental CD133 percentage more efficiently than purified CD133^−^ cells (data not shown). Also, it has also been reported that CD133^+^ ARO cells undergo asymmetric division and give rise to both CD133^+^ and CD133^−^ progeny [16]. These findings indicate that in thyroid cancers, the CD133^+^ and CD133^−^ populations are in dynamic equilibrium during cancer progression. We characterize the CD133^+^ population as a more undifferentiated and radioresistant population, and suggest that the proportion of CD133^+^ cells within the thyroid cancer population may determine the degree of malignancy of the cancer. Whether CD133 expression can serve as a prognostic factor should be intensively investigated. Several clinical trials have used retinoic acid, bexarotene, valproic acid, suberoylanilide hydroxamic acid or isotretinoin to reinduce the susceptibility of thyroid cancer to ^131^I therapy, the concept of “redifferentiation” therapy [31, 32]. These agents preferentially enhance the expression of thyroid-specific genes including NIS, and lead to the restoration of radioiodide uptake. It remains to be elucidated whether these agents have a differentiation effect on the CD133^+^ population or CSCs in thyroid cancer. Nevertheless, CD133-expressing thyroid cancer cells may be a potential target for thyroid cancer treatment.

### Conclusion

In summary, this study demonstrated the role of CD133-expressing cells in the therapeutic resistance of thyroid cancers. This specific population detected in thyroid cancers exhibited undifferentiated, radioresistance and survived ^131^I therapy. These findings provide a new perspective in the therapy of thyroid cancer and point to targeting CD133-expressing cells in the development of a therapeutic strategy.
